# Serum proteome profiles revealed dysregulated proteins and mechanisms associated with fibromyalgia syndrome in women

**DOI:** 10.1038/s41598-020-69271-w

**Published:** 2020-07-23

**Authors:** Chia-Li Han, Yung-Ching Sheng, San-Yuan Wang, Yi-Hsuan Chen, Jiunn-Horng Kang

**Affiliations:** 1 Master Program in Clinical Pharmacogenomics and Pharmacoproteomics, College of Pharmacy, Taipei Medical University, Taipei, 11031 Taiwan; 2 Department of Chemistry, National Taiwan University, Taipei, 10617 Taiwan; 3 Department of Physical Medicine and Rehabilitation, Taipei Medical University Hospital, Taipei, 11031 Taiwan; 4 Department of Physical Medicine and Rehabilitation, School of Medicine, College of Medicine, Taipei Medical University, 250 Wuxing St., Taipei, 11031 Taiwan; 5 Research Center of Artificial Intelligence in Medicine, Taipei Medical University, Taipei, 11031 Taiwan

**Keywords:** Inflammation, Biomarkers

## Abstract

Fibromyalgia syndrome (FM) is a multifactorial disorder whose pathogenesis and diagnosis are poorly understood. This study investigated differential serum proteome profiles in patients with FM and healthy pain-free controls and explored the association between serum proteome and clinical profiles in patients with FM. Twenty patients with FM (according to the American College of Rheumatology criteria, 2010) and 20 healthy pain-free controls were recruited for optimized quantitative serum proteomics analysis. The levels of pain, pressure pain threshold, sleep, anxiety, depression, and functional status were evaluated for patients with FM. We identified 22 proteins differentially expressed in FM when compared with healthy pain-free controls and propose a panel of methyltransferase-like 18 (METTL18), immunoglobulin lambda variable 3–25 (IGLV3–25), interleukin-1 receptor accessory protein (IL1RAP), and IGHV1OR21-1 for differentiating FM from controls by using a decision tree model (accuracy: 0.97). In addition, we noted several proteins involved in coagulation and inflammation pathways with distinct expression patterns in patients with FM. Novel proteins were also observed to be correlated with the levels of pain, depression, and dysautonomia in patients with FM. We suggest that upregulated inflammation can play a major role in the pathomechanism of FM. The differentially expressed proteins identified may serve as useful biomarkers for diagnosis and evaluation of FM in the future.

## Introduction

Fibromyalgia syndrome (FM), manifesting as chronic widespread pain throughout the body, is associated with long-term pain and impaired quality of life, which can result in tremendous medical and socioeconomic burden^1–4^. The prevalence of FM was reportedly between 0.2 and 6.6%, predominantly affecting women in the general population^5^. The diagnosis of FM continues to be based mainly on clinical history taking and patients’ complaints. Currently, no specific laboratory measure or specific biomarkers are available for the diagnosis of FM.

FM has been recognized as a multifactorial disorder involving genetic, biological, and environmental factors. Although the pathogenesis of FM is poorly understood, altered central pain processing and pain sensitization are key elements in the pathogenesis of FM^2,6–8^. Neuroimaging and neuroendocrine research have revealed that altered central nervous system structure and function occur in patients with FM^9–11^. Patients with FM also exhibit a wide spectrum of associated symptoms such as sleep disturbance, depression, anxiety, fatigue, other pain disorders, and cognition problems^12^. These symptoms add significant heterogeneity and difficulty in the clinical management of FM. Furthermore, the linkage of relevant biological pathways with clinical symptom profiles is poorly understood. Therefore, FM treatments currently available are primarily symptomatic and usually require a multidisciplinary approach^13^. Exploration of potential biomarkers that correlate with the clinical profiles of FM can be valuable for evaluating the patients with FM.

Proteomics analysis based on modern liquid chromatography–mass spectrometry (LC–MS) has enabled systematic profiling of proteome expressions in various specimens to identify disease-associated proteins. A few proteomics studies on FM have identified potential protein biomarkers in saliva^14,15^ and serum or plasma^16,17^. Proteomics analyses of the cerebrospinal fluid (CSF) in FM revealed dysregulation of proteins involved in lipoprotein lipase activity, inflammatory signaling, energy metabolism, and neuropeptide signaling^18^ as well as of proteins related to pain^19^. However, none of the respective candidates were replicated in salivary and blood proteomics studies. Moreover, CSF proteins are not ideal for clinical diagnosis. More extensive proteomics analysis in easily accessible specimens for FM detection is required. For this purpose, the present study investigated differential serum proteome profiles in patients with FM and healthy pain-free controls by using an optimized serum proteomics analysis workflow. We further explored the underlying molecular mechanism and the correlation between serum proteome and clinical profiles in patients with FM. These data can clarify the FM pathogenic mechanism and can help provide insights to objectively evaluate patients with FM and to develop specific treatments for FM.

## Results

### Optimization of depletion of high-abundance serum proteins prior to proteomics analysis

In this study, we adopted the commercially available multiple affinity removal system (MARS) Hu-14 column, which utilizes antibodies targeting the top 14 high-abundance serum proteins to selectively remove high-abundance proteins and generate depleted serum samples for proteomics analysis. The SDS-PAGE analysis of raw and depleted serum samples in Supplementary Fig. S1A revealed a significant depletion of high-abundance proteins and enhanced detection of other protein bands by using the MARS Hu-14 column. We further evaluated the loading amount for the MARS column; two loading conditions, a fixed serum volume (according to manufacturer’s instructions), and a fixed amount of serum proteins were tested using three selected serum samples with low (40.7 μg/μL), medium (70.9 μg/μL), and high (126.9 μg/μL) protein concentration levels. The depleted serum samples were analyzed in duplicate by using LC–MS/MS to obtain relative protein mass spectrometry (MS) abundances for comparison. As shown in Supplementary Fig. S1B, the use of fixed serum volume caused unequal depletion efficiency in the MARS column; the total MS abundances of the top 14 high-abundance proteins in the depleted serum samples were 34%, 26%, and 10% in high-, medium-, and low-concentration serum samples, respectively. The unequal depletion of high-abundance proteins led to significant bias in the precise quantitation of mid-to-low abundance proteins. By contrast, the use of a fixed serum protein quantity resulted in a more consistent depletion efficiency in the MARS column for high-abundance proteins (19–22%). We also tested various fixed quantities of serum proteins for MARS depletion. As seen in Supplemental Fig. S1C, an increase to 750 or 800 μg of serum proteins for the MARS column generated a similar depletion efficiency. Based on these observations and the resulting quantity of depleted proteins required for proteomics analysis, we selected the fixed quantity of 750 μg of serum proteins as preliminary materials for quantitative serum proteomics analysis.

### Quantitative profiling of serum proteome identified dysregulated proteins and mechanisms associated with FM

A total of 20 patients with FM and 20 healthy pain-free controls were recruited for quantitative serum proteomics analysis. The demographic data and clinical profiles are summarized in Table 1. The body mass index (BMI), work, and marital status were similar for patients with FM and controls. The age of patients with FM was slightly higher than that of controls (47.50 ± 7.45 vs. 52.90 ± 9.58 years, P < 0.048). Patients with FM experienced pain for an average of 9.05 years, significantly poorer sleep quality, and more severe anxiety and depression (P < 0.01). In addition, patients exhibited abnormally elevated levels for the widespread pain index (WPI; 7.35 ± 4.77), symptom severity scale (SSS; 5.05 ± 2.46), fibromyalgia impact questionnaire (FIQ; 43.53 ± 12.70), visual analog scale (VAS; 5.00 ± 2.18), and tender point pressure (2.35 ± 1.04) kg/cm^2^.

**Table 1:** Characteristics and clinical data of patients with FM and healthy pain-free controls.

	Control group (n = 20)	FM group (n = 20)	P value
Age, mean ± SD years	47.50 ± 7.45	52.90 ± 9.58	0.048
BMI, mean ± SD	21.62 ± 3.28	22.83 ± 4.01	0.204
Pain duration, mean ± SD years	0 ± 0	9.05 ± 8.89	< 0.01
Work status, n (%)			0.784
Employed	18 (90)	16 (80)	
Unemployed	2 (10)	4 (20)	
Marital status, n (%)			0.143
Single	16 (80)	15 (75)	
Married	4 (20)	5 (25)	
PSQI	3.95 ± 1.43	10.40 ± 3.03	< 0.01
BAI	2.74 ± 3.86	13.85 ± 8.75	< 0.01
BDI	3.25 ± 4.23	14.00 ± 9.78	< 0.01
WPI	–	7.35 ± 4.77	–
SSS	–	5.05 ± 2.46	–
FIQ	–	43.53 ± 12.70	–
VAS	–	5.00 ± 2.18	–
PPT, mean ± SD kg/cm^2^	–	2.35 ± 1.04	–

*BMI* Body Mass Index, *PSQI* Pittsburgh Sleep Quality Index, *BAI* Beck Anxiety Inventory, *BDI* Beck Depression Inventory, *WPI* Widespread Pain Index, *SSS* Symptom Severity Scale, *FIQ* Fibromyalgia Impact Questionnaire, *VAS* Visual Analogue Scale, *PPT* Pressure Pain Threshold, *P value* Mann–Whitney U Test for continuous variables and chi-square test for categorical variables.

**Figure 1:**
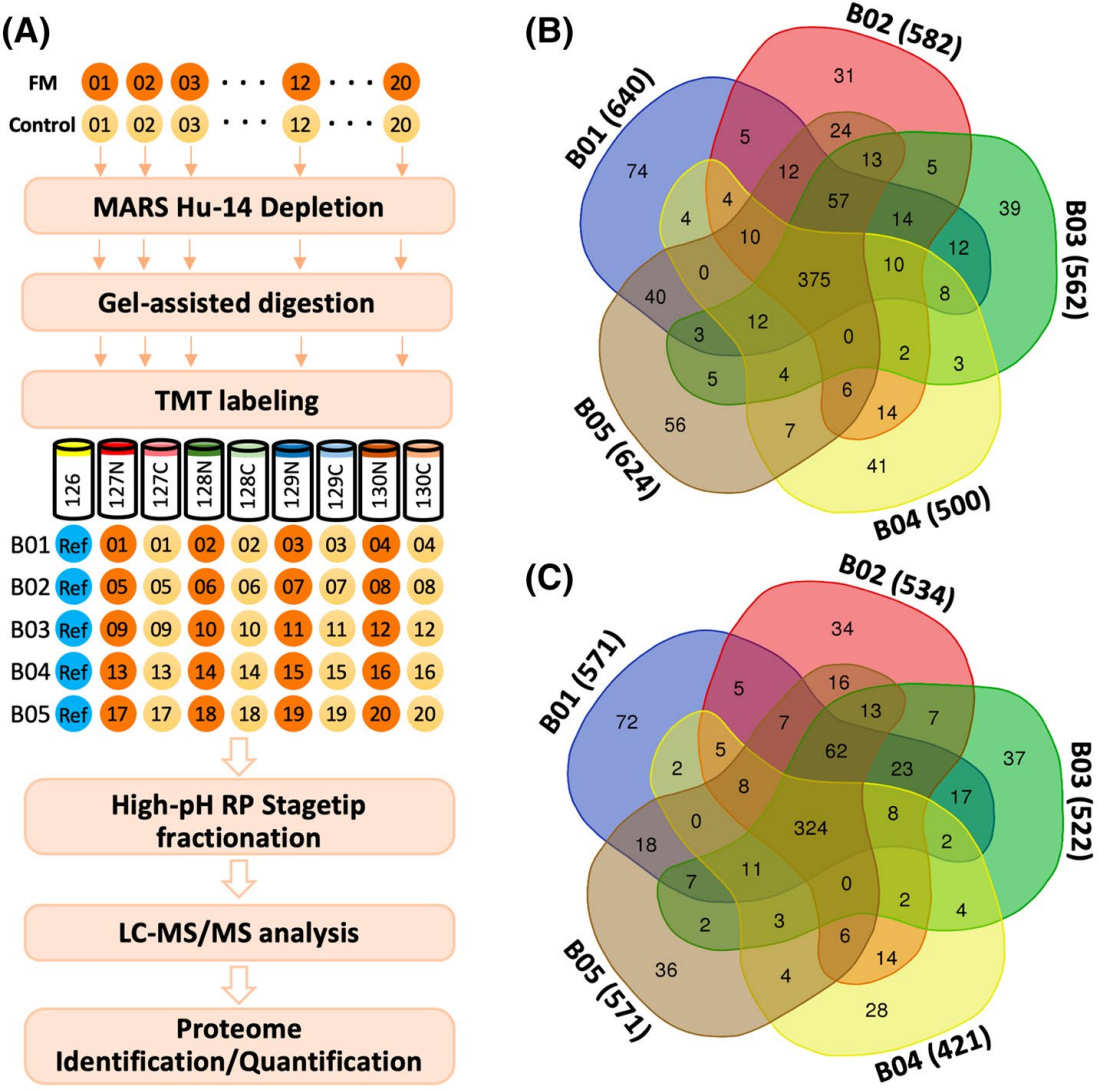
(**A**) Experimental workflow for FM serum proteome analysis. The overlapping of (**B**) identified and (**C**) quantified proteins in the five batches of TMT experiments.

To achieve in-depth profiling of serum proteome, we used a tandem mass tag (TMT)-based quantitation strategy, which integrated optimized MARS Hu-14 depletion, gel-assisted digestion, TMT tagging, high-pH reversed-phase (Hp-RP) StageTip fractionation, and LC–MS/MS analysis (Fig. 1A). Five batches of TMT experiments were performed using 40 samples and control references, which identified 890 serum proteins. Across 40 samples, 375 proteins were commonly identified (Fig. 1B), of which 324 were successfully quantified (Fig. 1C). A total of 28–72 proteins were uniquely quantified in one of the batches, suggesting heterogeneous expression of serum proteins among individuals.

**Figure 2:**
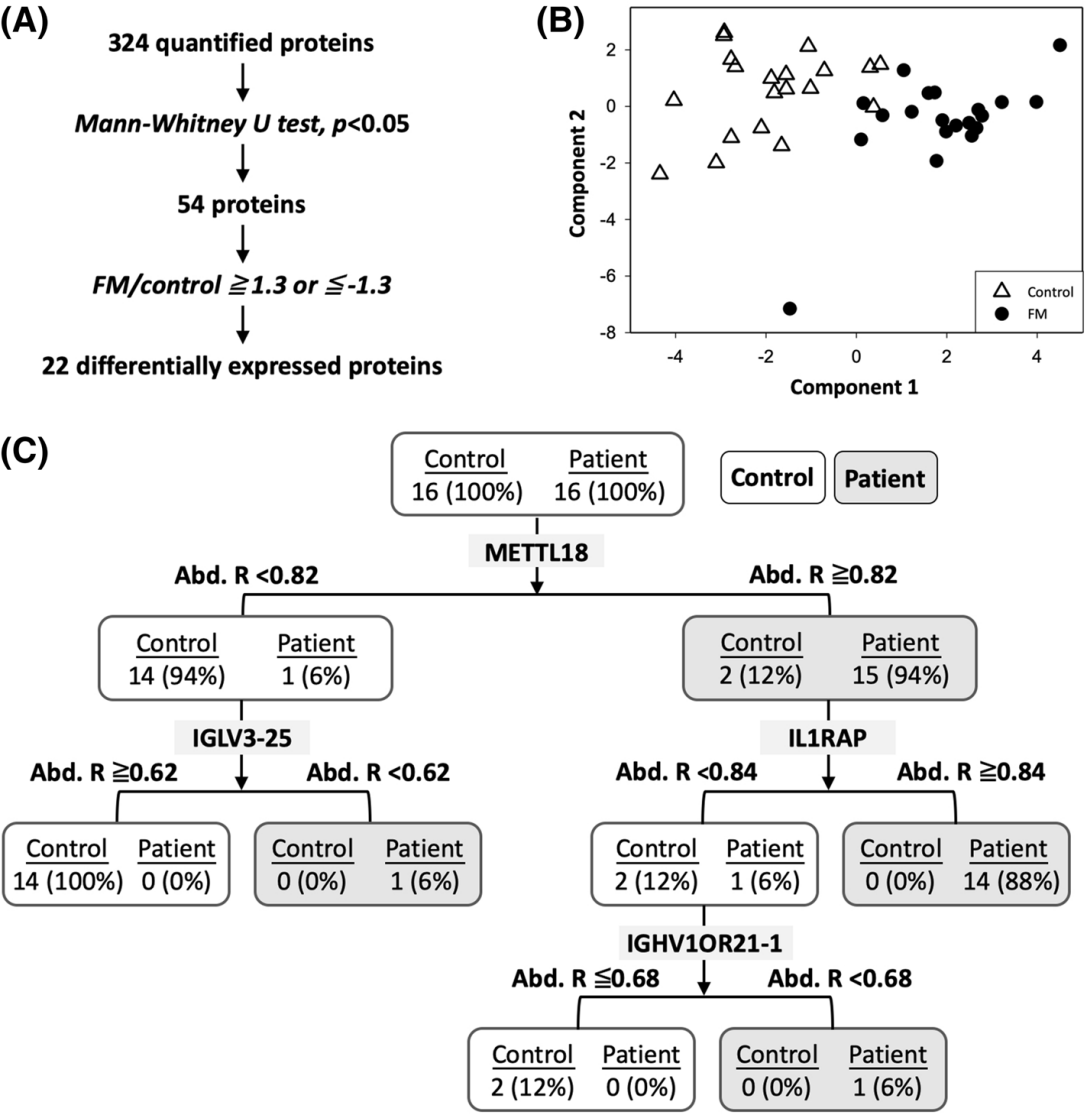
(**A**) The analysis workflow for filtering significant candidate proteins. (**B**) The PLS-DA plot shows a clear grouping of patients with FM and controls by using the 22 candidate proteins. The PLS-DA transformation preserves as much covariance as possible between the 22 candidate proteins and sample labels in the first component, which is the most relevant for distinguishing sample labels. The first two components are retained to distinguish the control and FM samples. (**C**) The decision tree analysis of candidate proteins suggests a panel of four proteins for accurate identification of patients with FM.

We next filtered candidate proteins from 324 commonly quantified proteins by using the Mann–Whitney U test (Fig. 2A). Only proteins with P values of < 0.05 and fold changes of > 1.3 or < − 1.3 were considered significant candidates for distinguishing between patients with FM and healthy pain-free controls. Based on these criteria, 22 proteins were selected as candidate proteins (Table 2), of which 9 and 13 proteins were upregulated and downregulated, respectively, in patients with FM compared with controls. We further applied partial least squares discriminant (PLS-DA) analysis for the 22 candidate proteins. The PLS-DA transformation preserved as much covariance as possible between the 22 candidate proteins and sample labels in the first component, which is the most relevant for distinguishing sample labels. The first two components were retained to distinguish control and FM samples. As shown in Fig. 2B, the PLS-DA analysis of the 22 candidate proteins revealed a clear distinction between patients with FM and controls.

**Table 2:** Significant candidate proteins in FM.

Accession	Gene symbol	Protein name	MS abundance in patients with FM	MS abundance in healthy controls	Fold change (FM/control)	VIP	P value^#^
**(A) Upregulated in FM**							
Q86YZ3	HRNR	Hornerin	1.026 ± 1.485	0.502 ± 0.173	2.04	0.63	**
O95568	METTL18	Histidine protein methyltransferase 1 homolog	1.055 ± 0.219	0.660 ± 0.175	1.60	1.69	***
Q6KB66	KRT80	Keratin, type II cytoskeletal 80	1.112 ± 1.067	0.749 ± 0.209	1.49	0.57	*
P02743	APCS	Serum amyloid P-component	1.100 ± 0.383	0.788 ± 0.390	1.40	0.88	**
P0C0L4	C4A	Complement C4-A	1.129 ± 0.375	0.851 ± 0.354	1.33	0.96	*
Q9NPH3	IL1RAP	Interleukin-1 receptor accessory protein	1.128 ± 0.226	0.856 ± 0.240	1.32	1.18	***
Q9BYE2	TMPRSS13	Transmembrane protease serine 13	1.096 ± 0.159	0.841 ± 0.118	1.30	1.61	***
O75015	FCGR3B	Low affinity immunoglobulin gamma Fc region receptor III-B	1.246 ± 0.353	0.957 ± 0.473	1.30	0.77	*
P08637	FCGR3	Low affinity immunoglobulin gamma Fc region receptor III-A	1.246 ± 0.354	0.957 ± 0.473	1.30	0.77	*
**(B) Downregulated in FM**							
P35542	SAA4	Serum amyloid A-4 protein	0.906 ± 0.195	1.181 ± 0.381	− 1.30	0.97	*
P01717	IGLV3-25	Ig lambda chain V-IV region Hil	0.936 ± 0.991	1.224 ± 0.528	− 1.31	0.73	***
P40197	GP5	Platelet glycoprotein V	0.895 ± 0.258	1.176 ± 0.281	− 1.31	1.07	***
P00734	F2	Prothrombin	0.919 ± 0.331	1.207 ± 0.581	− 1.31	0.79	*
P01743	IGHV1-46	Ig heavy chain V-I region HG3	0.856 ± 0.731	1.137 ± 0.655	− 1.33	0.81	*
A6NJS3	IGHV1OR21-1	Putative V-set and immunoglobulin domain-containing-like protein	0.850 ± 0.732	1.150 ± 0.695	− 1.35	0.82	*
P23083	IGHV1OR15-1	Ig heavy chain V-I region V35	0.850 ± 0.732	1.150 ± 0.695	− 1.35	0.82	*
P0CG05	IGLC2	Ig lambda-2 chain C regions	0.862 ± 0.609	1.172 ± 0.518	− 1.36	0.76	*
P35442	THBS2	Thrombospondin-2	0.898 ± 0.295	1.232 ± 0.346	− 1.37	1.13	***
P02671	FGA	Fibrinogen alpha chain	0.867 ± 0.490	1.201 ± 0.464	− 1.39	0.81	*
P07359	GP1BA	Platelet glycoprotein Ib alpha chain	0.847 ± 0.169	1.193 ± 0.333	− 1.41	1.29	**
P07737	PFN1	Profilin-1	0.900 ± 0.205	1.336 ± 0.563	− 1.49	1.07	**
P07996	THBS1	Thrombospondin-1	0.786 ± 0.396	1.250 ± 0.558	− 1.59	1.01	**

*VIP* Variable importance in the projection, *P value* Obtained with the Mann–Whitney U test.

^#^P < 0.05: *; P < 0.01, **; P < 0.005, ***.

These 22 candidate proteins were mainly involved in biological processes such as blood coagulation, immune response, and extracellular matrix–receptor interactions. The functional enrichment analysis performed using the ingenuity pathways analysis (IPA) indicated several altered pathways, including acute phase response signaling, liver X receptor–retinoid X receptor (LXR–RXR) activation, and synaptogenesis signaling pathways (Supplementary Table S1). In addition, the upstream regulator analysis suggested that the levels of tumor necrosis factor-α (TNF-α) and transforming growth factor-β1 (TGFB1) were higher and those of interleukin (IL)-6, lipopolysaccharides, and MYC were lower in patients with FM than in controls (Supplementary Table S2). IL6, an inflammatory cytokine, is also a primary regulator of fibrinogen synthesis. The coordinate downregulation of fibrinolysis proteins (F2, GP5, FGA, GP1BA, THBS1, and THBS2) in our data suggests a lower level of IL-6 in patients with FM.

### Decision tree analysis identified a panel of protein candidates for fibromyalgia detection

To determine the diagnostic potential of these candidate proteins, we applied decision tree analysis for the abundance ratio of the 22 candidate proteins to create a protein panel for FM detection. According to the Pareto Principle, known as the 80–20 rule, the ratio of 80:20 is commonly used for training and validation of data sets in supervised learning algorithms, namely decision tree algorithms^20^. Hence, we used 80% of data to train and construct a decision. The remaining 20% of data were used to validate the efficiency of the decision tree. As seen in Fig. 2C, a panel of METTL18 (abundance ratio ≥ 0.82), IGLV3-25 (abundance ratio < 0.62), IL1RAP (abundance ratio ≥ 0.84), and IGHV1OR21-1 (abundance ratio < 0.68) offered 100% detection sensitivity for patients with FM in the training cohort. Even a single protein, METTL18, could achieve a detection sensitivity of 0.94 and a precision of 0.88 (Table 3). The panel was further verified in the validation cohort. All combinations of protein panels yielded identical performances in terms of sensitivity (1.0) and specificity (0.88) for the detection of FM (Table 3).

**Table 3:** Performance of different protein panels for FM detection.

Candidate	Training cohort (16 patients with FM and 16 controls)				Validation cohort (4 patients with FM and 4 controls)			
	Accuracy	Sensitivity	Precision	F-score	Accuracy	Sensitivity	Precision	F-score
METTL18	0.91	0.94	0.88	0.9	0.88	1	0.88	0.89
METTL18 + IGLV3-25	0.94	1	0.89	0.94	0.88	1	0.88	0.89
METTL18 + IL1RAP	0.94	0.88	1	0.94	0.88	1	0.88	0.89
METTL18 + IGLV3-25 + IL1RAP	0.94	0.88	1	0.94	0.88	1	0.88	0.89
METTL18 + IL1RAP + IGHV1OR21-1	0.97	0.94	1	0.97	0.88	1	0.88	0.89

### Clinical correlation of differential serum proteome profiles in fibromyalgia

**Figure 3:**
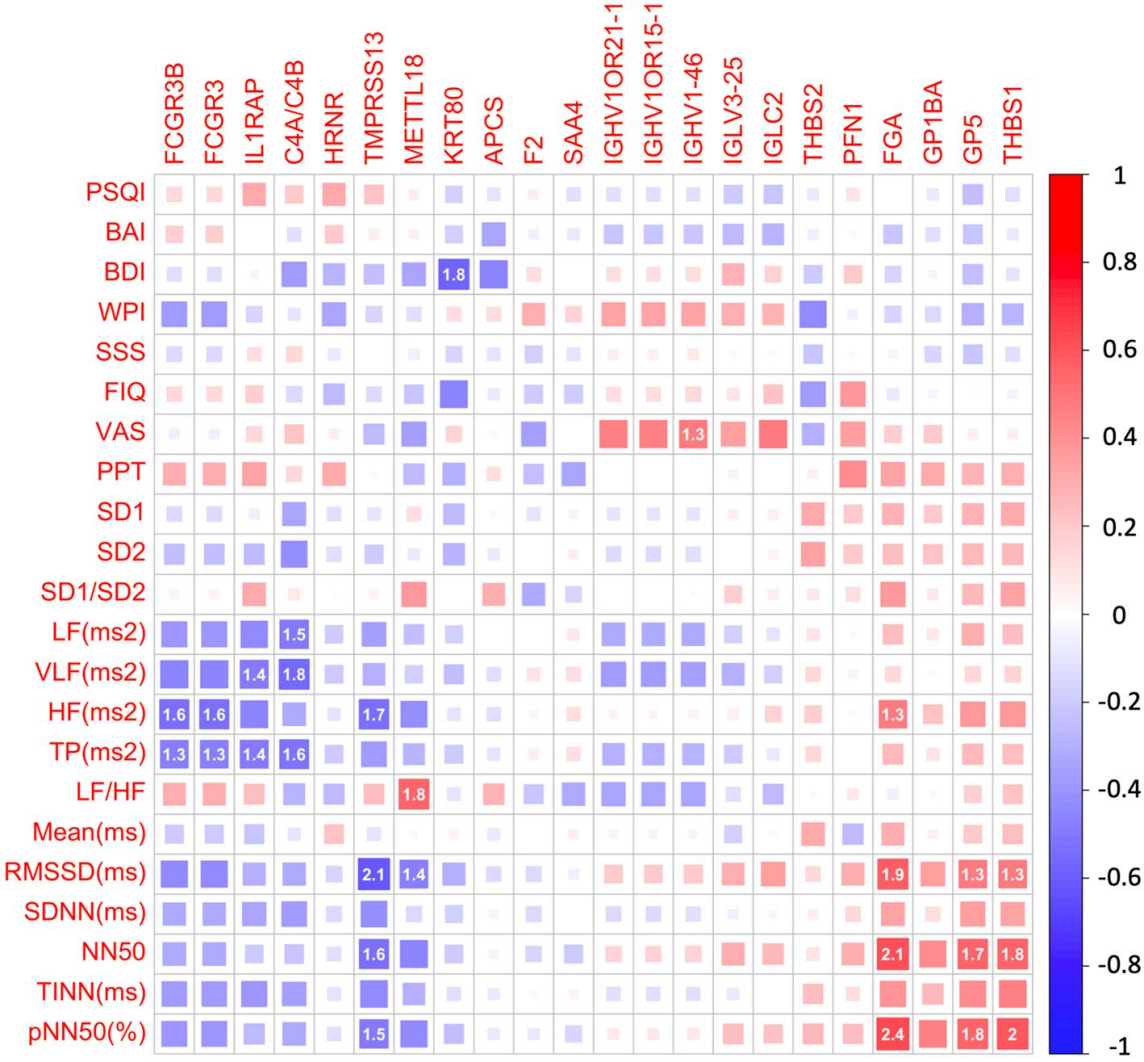
The partial correlation among 22 candidate proteins, clinical symptoms, and HRV parameters. The color of the box indicates the correlation of candidate proteins and clinical data. The size of the box indicates the − log(P value). Only the correlation with P < 0.05 was labeled with the − log(P value) value in the box. *VAS* Visual analog scale of pain, *PPT* pressure pain threshold, *FIQ* Fibromyalgia impact questionnaire, *PSQI* Pittsburgh sleep quality Index, *BDI-II* Becker’s depression inventory version II, *BAI* Becker’s anxiety inventory, *HRV* heart rate variability. Parameters in HRV include *SD1* standard deviation 1 of the scattergram, *SD2* standard deviation 2 of the scattergram, *SD1–SD2* ratio of SD1 to SD2, *LF (ms2)* low frequency, *VLF (ms2)* very low frequency, *HF (ms2)* high frequency, *TP (ms2)* total power, *LF–VLF* low-frequency energy to high-frequency energy, *RMSSD (ms)* root mean square difference of successive normal R–R intervals, *SDNN (ms)* Standard deviation of NN intervals, *NN50* Number of pairs of successive NNs that differ by more than 50 ms, *TINN (ms)* Triangular interpolation of the NN interval histogram, *pNN50(%)* Proportion of NN50 divided by total number of NNs

We conducted a partial correlation analysis among filtered candidate proteins, clinical symptoms, and heart rate variability (HRV) parameters (Fig. 3). For symptom profiles, the expressed levels of IGHV1-46 were slightly correlated with VAS for pain (P = 0.0493, r = 0.4696) and the level of KRT80 was significantly correlated with the score of BDI (P = 0.0142, r = − 0.5666). For HRV parameters, the expression level of C4 was significantly correlated with total power, low frequency (LF) power (P = 0.0332, r = − 0.5035), and very-low-frequency (VLF) power (P = 0.0162, r = − 0.5577). The level of TMPRSS13 was correlated with root mean square successive differences (RMSSDs; P = 0.0075, r = − 0.6071) and the number of pairs of successive normal-to-normal beats (NNs) differing by more than 50 ms (NN50; P = 0.0238, r = − 0.5297). The level of METTL18 was correlated with RMSSD (p = 0.0429, r = − 0.4817) and low frequency–high frequency (LF–HF) ratio (P = 0.0165, r = 0.5563); the expression level of FGA was correlated with HF (P = 0.0473, r = 0.5563), RMSSD (P = 0.0140, r = − 0.4817), and NN50 (P = 0.0071, r = − 0.4565). The level of GP5 was correlated with RMSSD (P = 0.455, r = 0.4766) and NN50 (P = 0.0192, r = 0.5455). The level of THBS1 was correlated with RMSSD (P = 0.457, r = 0.4762), and NN50 (P = 0.0169, r = 0.5548). The expression level of FCGR3B was correlated with HF (P = 0.0239, r = − 0.5294) and TP (P = 0.0449, r = − 0.4778).

## Discussion

Among the possible factors that underlie FM pathophysiology, a proinflammatory status and a decreased antioxidant capacity are commonly reported in patients with FM^21,22^. However, these changes are difficult to use as clinical biomarkers because most of them lack specificity. One advantage of using proteomics to investigate potential biomarkers of FM is the ensemble feature of proteomics. The novelty of the present study is using machine learning to explore the complex proteomics and locate potential biomarkers in patients with FM. Using a decision tree model, we could successfully differentiate patients with FM from controls based on the expression levels of METTL18, IGL3-25, and IL1RAP, yielding an accuracy of up to 0.97. We developed a diagnostic panel and decision tree by using a combination of more than two candidate proteins, which yielded better specificity and sensitivity in the diagnosis of FM. This panel may serve as an objective diagnostic tool for FM in the future. However, it is difficult to claim the proteomic difference exhibited in present study is associated with FM or chronic pain. Further study to compare the proteomic profile between FM and patients with other well-characterized chronic pain disorders should be considered.

We found that METTL18, known as histidine protein methyltransferase 1 homolog, is an efficient candidate protein for differentiating patients with FM from controls. However, data regarding the biological function of METTL18 are scant. The most well-known protein database, UniProt, suggests that METTL18 acts as a protein methyltransferase and participates in fundamental protein modification and heat shock protein binding. Nevertheless, the role of METTL18 in FM pathophysiology requires further study. A significant downregulation of immunoglobulin G-associated proteins (IGLV3-25, IGHV1-46, IGHV1OR21-1, IGHV1OR15-1, and IGLC2) was noted in the present study. Although relevant data are scant, an unexpectedly high prevalence of immunoglobulin deficiency in FM was reported^23^. Furthermore, FM is common in patients with primary immunodeficiency^24^. Primary immune deficiency states are well known to predispose patients to autoimmunity. This finding reinforces the hypothesis that FM is associated with dysregulated immune response.

After performing further pathway analysis for candidate proteins (Supplementary Table S1), we observed that proteins (complement C4-A, IL-1 receptor accessory protein, and immunoglobulin gamma Fc region receptor III-A and B) and pathways involved in coagulation and inflammation were affected mainly in patients with FM. Furthermore, we found that proteins involved in the activation of the retinoid X receptors and the liver X receptor involved in the regulation of inflammation response showed increased activation from the IL-1 to the Nf-kB pathway in patients with FM. Ramírez-Tejero et al*.* reported the plasma proteomic signature in patients with FM and also found differentially expressed proteins to be mainly involved in inflammatory (LXR–RXR) and coagulation pathways^16^. Our results suggest that inflammation plays a role in the pathophysiology of patients with FM, as seen in another study^25^. Subtyping of FM according to unique inflammatory features as inflammatory FM has been proposed^26^. The role of inflammation in FM pathogenesis, as least in certain subtypes, should be further studied.

In one large cross-sectional study, serum CRP, a proinflammatory systemic biomarker, was significantly elevated in patients with FM^27^. In that study, notably, the mean level of CRP remained within normal reference values in patients with FM. This finding implies modest systemic inflammation in patients with FM. For the exploration of specific inflammatory responses involved in FM, many studies have reported altered cytokine patterns in the blood and CSF of patients with FM, but the results are varied and inconsistent^28–31^. Inflammation is regulated through a complex network of interactions among proinflammatory and anti-inflammatory pathways^32^. A dysregulated proinflammatory and anti-inflammatory response may be an underlying cause of FM^31,33^. Increased release of IL-1 beta, TNF-α, IL-6, IL-8, and IL-10 from stimulated monocytes in FM was reported^34^, although this remains controversial^29^. Abnormal release of cytokines from peripheral immune cells may be associated with fatigue, hyperalgesia, and allodynia in FM^35^. A meta-analysis concluded that patients with FM have elevated blood levels of IL-1 receptor antagonist (IL-1ra), IL-6, and IL-8^28^. Studies have suggested that serum IL-6 is elevated in patients with FM and is correlated with disease severity^28,29^. In contrast with present data, we observed decreased IL-6 upstream activation based on candidate proteins’ expression (Supplementary Table S2). A few studies have reported elevated levels of proinflammatory cytokines, such as IL-8, monocyte chemoattractant protein-1, and IL-17A,^36,37^ but decreased levels of anti-inflammatory cytokines, such as IL-4 and IL-13^38^. These findings reflect the complexity and heterogeneity in the pathophysiology of FM. The discrepancy in results may be explained by variations in cytokine profiles in a brief time and in different disease statuses (acute vs. chronic) of FM^39^. The inconsistency in results might also be due to the use of different types of body fluids and assays.

Neuroinflammation in CNS has been suggested to be involved in the FM pathogenesis. Evidence shows the potential linkage of coagulation and fibrinolysis at the neurovascular interface to neuroinflammation and degeneration^40^. Fibrin, as a final product in the coagulation cascade appearing at the neurovascular interface, could be associated with further inflammatory responses, including immune cell migration to the brain. Dysregulation of the coagulation and fibrinolysis system has been observed in several CNS diseases, such as multiple sclerosis and Alzheimer’s disease^41,42^. In the present study, we found alterations in the regulation of several coagulation and fibrinolysis factors, including platelet glycoprotein Ib alpha chains, thrombospondin-1, platelet glycoprotein V, prothrombin, thrombospondin-2, fibrinogen alpha chains, and platelet glycoprotein Ib alpha chains, in patients with FM. Other studies have seldom mentioned the altered coagulation function in patients with FM. Future studies should explore the pathogenic effects of altered coagulation on symptoms or neuroinflammation in patients with FM.

Currently, the pharmacological approach for FM treatment focuses primarily on neuromodulation (anticonvulsants and antidepressants). Nonsteroidal anti-inflammatory drugs and steroids targeting anti-inflammation yielded inconsistent results in the treatment of FM^43,44^. For clinical applications, more specific treatments targeting inflammation may be considered for FM management. We found that TNF-α may be a potential upstream activation molecule in patients with FM (Supplementary Table S2). TNF-α is a pleiotropic mediator of physiological and neurological functions. In pathological brains, research showed that TNF-α exerted a neuroinflammatory and neurotoxic effect^45^. One study showed that abnormal activation of microglia in FM was reportedly likely to exhibit hypersensitivity and overproduction of TNF-α in response to stimulation^46^. Another study showed that obesity-induced TNF-α release may potentiate FM-associated pain in mouse models^47^. We also found that inflammation-associated LXR–RXR involving the IL-1 to Nf-kb pathway was activated in patients with FM. Activation of the IL-1 beta pathway has been observed to be required for the development of mechanical allodynia in neuropathic pain^48^. Since there is no efficient treatment for FM currently, treatments aim to these dysregulated pathways may be further explored.

The subtyping of FM into phenotypes according to pain, sleep, and psychological symptom profiles has been suggested^21,22^. These FM subtypes may specifically involve different underlying biological pathways. Using this concept, we analyzed the correlation between serum protein expression patterns and symptom profiles in patients with FM. We found that the expression level of the Ig heavy chain V-I region HG3 was positively correlated with pain intensity in patients with FM. Growing evidence suggests that immunoglobulin therapy can be a potential treatment for chronic pain, including FM, complex regional pain syndrome, and pain associated with specific autoantibodies^49^. The analgesic immunoglobulin G might be associated with the modulation of cytokine expression and function^50^.

The level of type II cytoskeletal 80 was negatively correlated with the depression level. The roles of these proteins in the symptom profiles of FM are of potential interest to researchers. A dysfunctional hypothalamic–pituitary–adrenal axis and dysautonomia are common in FM^51^. The level of dysautonomia was correlated with symptom severity in patients with FM. In this study, we found the level of FGA, a subunit of fibrinogen, to be significantly correlated with altered sympathetic–parasympathetic balance in HRV. Fibrinogen expression can reportedly differentiate patients with FM from controls in a proteomics study^16^. Fibrinogen is a protein involved in acute systemic responses to inflammation, stress, and coagulation. We suggest that fibrinogen levels can act as a surrogate marker to evaluate the level of dysautonomia in patients with FM. IL6 is also a primary regulator of fibrinogen synthesis. Intervention to IL-6 associated pathway may be a candidate strategy in managing dysautonomia in patients with FM.

The serum is an easily accessible clinical specimen and thus is ideal for disease detection. However, identification of potential protein biomarkers in serum by using MS-based proteomics analysis is extremely challenging because of the wide dynamic ranges of serum proteins and the masking effect of high-abundance proteins. To overcome these limitations, two-dimensional gel electrophoresis^16^ and label-free-based^17^ shotgun proteomics strategies were applied in serum or plasma samples to screen FM-associated proteins, enabling the identification of 266 proteins. The present study utilized the TMT-based proteomics approach and extensive peptide fractionation by Hp-RP StageTip, identifying 890 serum proteins, which is the largest serum proteome result in FM to date. Distinct serum proteomics profiles can be found in patients with FM compared with controls. Compared with approaches used in other studies, the approach used in the present study enabled a wider range of proteomic profiles to be investigated in patients with FM.

Limitations of the present study should be addressed. First, the sample size of the present study is relatively small. A large study is required to confirm our results. Second, recruited patients with FM and controls were all Han Chinese. Our results should be revisited in patients with FM of other ethnicities. Third, a cross-sectional study cannot identify the temporal relationship between symptoms and proteomics profiles. Interactions between proteins and biological pathways are complex, and primary and secondary pathophysiological changes in experimental participants cannot be differentiated in proteomics data. A longitudinal proteomics study in patients with FM may help clarify this. Fourth, because those affected by FM are predominantly women, the present study included only women to enhance the study population homogeneity. Finally, the symptomatic duration of patients with FM was not taken into account, because the syndrome onset time is often difficult to determine in patients with FM. Pathophysiological changes in FM have also been suggested to be associated with syndrome duration^25^.

In conclusion, patients with FM have differential serum proteomics pattern compared with healthy pain-free controls. We suggest that upregulated inflammation plays a major role in the pathogenesis of FM, as observed from the serum proteomics analysis. Combining the levels of METTL18, IGLV3-25, IL1RAP, and IGHV1OR21-1 can successfully differentiate FM patients from healthy pain-free controls. Differentially expressed proteins may serve as potential biomarkers for diagnosis and clinical evaluation of FM in the future.

## Methods

### Patient recruitment

Twenty women who fulfilled the American College of Rheumatology 2010 diagnostic criteria for FM were recruited from clinics in the university hospital^52^. We included women with FM aged > 20 years. The WPI was used to access the range of pain involved area. The SSS was used, including categorical scales for cognitive symptoms, unrefreshed sleep, fatigue, and a number of somatic symptoms. The diagnosis was established by a clinical specialist. Twenty healthy pain-free women were recruited as the control group. Each participant provided informed consent prior to inclusion. The study was approved by the Joint Institutional Review Board of Taipei Medical University (TMU-JIRB No.: N201702062). All methods herein were performed in accordance with relevant guidelines and regulations. The CONSORT flow diagram for participants’ recruitment was shown in Supplementary Fig. S2. Patients with the following conditions were excluded from this study:

1. Major psychiatric disorders, such as schizophrenia or schizoaffective disorders
2. Substance or alcohol abuse
3. Known concurrent malignancy
4. Major rheumatic diseases such as systemic lupus erythematous, Sjogren’s syndrome, rheumatoid arthritis, and ankylosing spondylitis
5. Pregnancy

### Questionnaire and measurements

For each patient with FM, measurements were taken by using the following scales:

1. Visual analog scale (VAS) of pain and pressure pain threshold (PPT)
2. Fibromyalgia impact questionnaire (FIQ)^53^
3. Pittsburgh sleep quality Index (PSQI)^54, 55^
4. Beck depression inventory version II (BDI-II)^56, 57^
5. Beck anxiety inventory (BAI)^58, 59^
6. Five-minute heart rate variability (HRV)

The details of these questionnaires and measurements are listed in Supplementary Information.

### Serum collection

To prevent interference from concurrent FM medications on proteomics analysis, patients with FM were asked to terminate use of medications for at least 2 weeks prior to serum collection. However, oral acetaminophen 3,000 mg/24 h was allowed for relief of pain when the patient was prevented from taking their usual pain medications during this period. Forty-milliliter blood samples from patients and controls were drawn in the early morning and after an overnight fast. The blood was disposed in ethylenediaminetetraacetic acid–free tubes and left undisturbed at ambient temperature for 30 min to allow clotting. The clot was removed through centrifugation at 1,300*g* for 10 min. After centrifugation, the serum supernatants were collected, aliquoted, and stored at − 80 °C until use.

### TMT-based quantitative serum proteomics analysis

Figure 1A illustrates the experimental workflow in this study. A total of 750 μg of serum proteins was aliquoted for the depletion of the top 14 high-abundance proteins by using a MARS Hu-14 column (Agilent Technologies, Waldbronn, Germany) according to the vendor’s protocol with several optimizations. After depletion, 50 μg of depleted serum proteins was subjected to gel-assisted digestion with trypsin individually^60^ to collect peptides for subsequent 10-plex TMT labeling (Thermo Fisher Scientific, San Jose, USA). For each TMT experiment, peptides from four FM patients were individually labeled with TMT_127N_, TMT_128N_, TMT_129N_, and TMT_130N_, while peptides from 4 controls were labeled with TMT_127C_, TMT_128C_, TMT_129C_, and TMT_130C,_ respectively. TMT_126_ were labeled with reference peptides from the pooling of 40 samples. Five batches of 10-plex TMT experiments were performed for 20 patients with FM and 20 healthy pain-free controls. In each batch, 9 TMT-labeled samples were combined for Hp-RP StageTip fractionation^61^ to generate six reversed-phase fractions, followed by duplicate analysis using LTQ Orbitrap Fusion mass spectrometers equipped with the Dionex Ultimate 3000 nanoLC system and a NanoSpray interface (Thermo Fisher Scientific). Protein identification and quantification were performed using Proteome Discoverer 2.1 (Thermo Fisher Scientific). The details of experiments, LC–MS/MS acquisition, and proteome identification or quantitation are included in Supplementary Information. The mass spectrometry proteomics data have been deposited in the jPOST repository^62^ with the dataset identifier PXD013905.

### Data processing, annotation, and statistical analysis

We used the Mann–Whitney U test to compare differences in demographic data and clinical profiles between controls and patients with FM as well as to evaluate the significance of protein fold changes between patients with FM and healthy pain-free controls^63^. The chi-squared test was used to determine the frequency difference in categorical variables between the two groups. PLS-DA^64^ was applied to visualize how efficiently significant candidates could distinguish patients from controls. The variable importance in the projection value of each protein in the PLS-DA model indicated its contribution to the classification of patients and controls. Eighty percent of patients with FM and healthy pain-free controls were randomly selected and used to train a decision tree by using the classification and regression tree analysis^65^ for distinguishing patients with FM from healthy pain-free controls and for developing decision rules for the diagnosis of FM. The remaining 20% of patients were used to validate decision rules.

The age and BMI of each patient were used as control variables to calculate the partial correlation between each significant protein fold change and the clinical data of patients with FM by using the R package “ppcor”^66^. All data processing and statistical analysis were performed using R (Version 3.5.1)^67^.

### Functional analysis

The significant candidate proteins in patients with FM were submitted for IPA (Qiagen Bioinformatics)^68^ for the enrichment analysis of dysregulated cellular functions, disease annotations, networks, pathways, and upstream regulators.

## Supplementary information

Supplementary Information.
