# Author Correction: Serum proteome profiles revealed dysregulated proteins and mechanisms associated with fibromyalgia syndrome in women

**DOI:** 10.1038/s41598-021-88212-9

**Published:** 2021-04-13

**Authors:** Chia‑Li Han, Yung-Ching Sheng, San‑Yuan Wang, Yi‑Hsuan Chen, Jiunn‑Horng Kang

**Affiliations:** 1 Master Program in Clinical Pharmacogenomics and Pharmacoproteomics, College of Pharmacy, Taipei Medical University, Taipei, 11031 Taiwan; 2 Department of Chemistry, National Taiwan University, Taipei, 10617 Taiwan; 3 Department of Physical Medicine and Rehabilitation, Taipei Medical University Hospital, Taipei, 11031 Taiwan; 4 Department of Physical Medicine and Rehabilitation, School of Medicine, College of Medicine, Taipei Medical University, 250 Wuxing St., Taipei, 11031 Taiwan; 5 Research Center of Artificial Intelligence in Medicine, Taipei Medical University, Taipei, 11031 Taiwan

Correction to: *Scientific Reports* 10.1038/s41598-020-69271-w, published online 23 July 2020

This Article contains errors in the Methods section under subheading ‘Patient recruitment’.

“The study was approved by the Joint Institutional Review Board of Taipei Medical University (TMU-JIRB No.: N201702062).”

should read:

“The study was approved by the Joint Institutional Review Board of Taipei Medical University (TMU-JIRB No.: N201701062 & N201812078).”

