# Supplementary material for: Serum proteome profiles revealed dysregulated proteins and mechanisms associated with fibromyalgia syndrome in women
Source: Sci Rep. 2020 Jul 23;10:12347. doi: 10.1038/s41598-020-69271-w (PMC7378543; doi:10.1038/s41598-020-69271-w)
Supplement: Supplementary file 1 — Supplementary Information. [file 41598_2020_69271_MOESM1_ESM.docx]

**Supplementary Information**

**Serum Proteome Profiles Revealed Dysregulated Proteins and Mechanisms Associated with Fibromyalgia in Women**

Chia-Li Han, PhD^1^, Yung-Ching Sheng, MSc^2&^, San-Yuan Wang, PhD^1&^, Yi-Hsuan Chen, BSc^1^, Jiunn-Horng Kang, MD, PhD^3,4,5*^

^1^ Master Program in Clinical Pharmacogenomics and Pharmacoproteomics, College of Pharmacy, Taipei Medical University, Taipei 11031, Taiwan

^2^ Department of Chemistry, National Taiwan University, Taipei 10617, Taiwan

^3^ Department of Physical Medicine and Rehabilitation, Taipei Medical University Hospital, Taipei 11031, Taiwan

^4^ Department of Physical Medicine and Rehabilitation, School of Medicine, College of Medicine, Taipei Medical University, Taipei 11031, Taiwan

^5^ Research Center of Artificial Intelligence in Medicine, Taipei Medical University, Taipei 11031, Taiwan

These authors contributed equally to this work

* Corresponding author

**Questionnaire and measurements**

**Fibromyalgia Impact Questionnaire**

The Fibromyalgia Impact Questionnaire (FIQ), a self-administered assessment instrument aims to measure the status and severity of symptoms in patients with FM. The FIQ measures the components of health and functional status that are generally affected by FM. The FIQ is composed of 20 items rating physical functioning, work difficulty, pain, fatigue, morning tiredness, stiffness, anxiety, and depression. The instrument takes approximately 5 minutes to complete. The FIQ is scored such that a higher score indicates that the syndrome affects the person more severely. Validity for FM effects is well documented with good psychometric properties. Currently, Chinese version of FIQ is still not available. We followed 4 steps to translate original version of FIQ to Chinese version in present study: forward translation, backward translation, expert discussion, and preliminary test.

**Pittsburgh Sleep Quality Index**

The Pittsburgh Sleep Quality Index (PSQI) is composed of 19 self-administered questions to measure sleep quality. The PSQI consists of seven components, and a global score is computed using the sum of scores from each component (in the range of 0–21). A global score of more than or equal to 6 indicates impaired sleep quality. The PSQI has been applied in several clinical and research contexts and demonstrates adequate psychometric properties for the evaluation of sleep quality. The validity of Chinese version of PSQI has been verified.

**Beck Depression Inventory, version II (BDI-II)**

The BDI-II is a 21-item self-report inventory, providing assessment and monitoring of depression severity. It is composed of items related to symptoms of depression, such as hopelessness and irritability; cognitions, such as guilt or feelings of being punished; and physical symptoms, such as fatigue, weight loss, and lack of interest in sex. The BDI-II has been tested for content, concurrent, and constructive validity. The validity of Chinese version of BDI-II has been verified.

**Beck Anxiety Inventory**

The BAI is a 21-question self-report inventory used for measuring the severity of anxiety in adults. The questions relate to common symptoms of anxiety that the respondent has experienced during the preceding week. Higher scores indicate more severe anxiety symptoms. Several studies have found the Beck Anxiety Inventory to be an accurate measure of anxiety symptoms in adults. The validity of Chinese version of BAI has been verified.

**Heart rate variability measurement**

Heart rate variability (HRV) can provide objective measurement regarding autonomic status. A 5-min continuous recording of a standard 3-lead electrocardiogram (ECG) was obtained in resting and sitting positions (MyECG, MSI, Taiwan) for controls and patients with FM. Each patient had to rest for at least 10 min prior to HRV measurement. The sampling frequency was set as 500 Hz. The raw data were extracted and further processed with MTALAB software. The raw ECG data were first visually checked by a clinician. If abnormal beats (arrhythmia) represented more than 5% of the total number of beats in a 5-min ECG recording, the ECG was excluded from HRV analysis. We used normal-to-normal beat intervals for HRV analysis. After detrending and filtering procedures were completed, the HRV was computed using both linear and nonlinear methods. The linear parameters were computed, including root mean square successive difference; the number of pairs of successive normal-to-normal beats (NNs) differing by more than 50 ms (NN50); the proportion of NN50 divided by the total number of NNs; the standard deviation of N–N intervals in the time domain; and the normalized power of LF, HF, VLF along with the LF–HF ratio in the frequency domain. Nonlinear parameters including sample entropy and Poincare plot were calculated.

**Pressure pain threshold test**

The patients’ pressure pain threshold (PPT) was assessed using a handheld pressure gauge (Algometer, Paintest, Wagner Inc, USA) to evaluate pain sensitivity in patients with FM. The assessment sites were the nine paired tender points of the diagnostic criteria for FM defined by ACR in 1990 and located at the occiput, lower cervical spine, trapezius, supraspinatus, second rib, lateral epicondyle, gluteus, greater trochanter, and knee. During measurement, the patient was in a relaxed sitting position. The investigator placed the pressure algometer on a site to be inspected and applied pressure in a vertical direction while increasing the force at a constant rate of approximately 1 kg/cm^2^. The investigator recorded values when the patient experienced only slight pain. The measurements were repeated three times and the results averaged. The unit of PPT is kg/cm^2^.

**Tandem mass tag-based quantitative serum proteome analysis**

**Depletion of high-abundant proteins in serum using Multiple Affinity Removal Spin Cartridge Human 14 (MARS Hu-14)**

The MARS Hu-14 column and buffers A and B were purchased from Agilent Technologies (Palo Alto, USA). We followed the vendor protocol for the MARS Hu-14 with several optimizations. The serum was filtered with a 0.22-µm spin filter and subjected to the bicinchoninic acid (BCA) assay (Thermo Fisher Scientific, San Jose, USA) to determine the crude serum protein concentration. Seven hundred and fifty micrograms of serum proteins was aliquoted and mixed with buffer A to obtain a final volume of 200 µL. One microliter of protease inhibitor cocktail was also added to each diluted sample. Before the diluted serum sample was loaded, the MARS Hu-14 column was washed and equilibrated sequentially with buffer A and buffer B according to the vendor’s protocol. The diluted serum sample was loaded onto the MARS Hu-14 column, which was placed in a 1.5-mL microcentrifuge tube and centrifuged at 100 ×*g* for 1 min at 4°C to collect the first flow-through; this was followed by the addition of 350 µL of buffer A to the MARS Hu-14 column and centrifuging at 100 ×*g* for 1 min at 4°C to obtain second flow-through. All of the flow-through was desalted using Amicon® Ultra3K Devices (Millipore, Bedford, MA, USA), combined with depleted serum proteins, vacuum dried using SpeedVac (EYELA, Tokyo, Japan), resuspended with 25 mM triethylammonium bicarbonate (TEABC, pH 8.5, Sigma-Aldrich), and subjected to the BCA assay to determine the protein concentration. The MARS Hu-14 column was washed three times using buffer B and balanced using buffer A twice, and the next sample was then loaded to the MARS Hu-14 column to deplete the top 14 high-abundance proteins.

**Gel-assisted digestion**

After MARS Hu-14 depletion, 50 µg of depleted serum proteins was aliquoted for our gel-assisted digestion. Proteins were reduced and alkylated using 5 mM of tris(2-carboxyethyl)phosphine and 2 mM methyl methanethiosulfonate, respectively. The protein solution was polymerized into gel by adding 40% acrylamide–bisacrylamide (29:1), 10% ammonium persulphate, and tetramethylethylenediamine. The gel was cut into pieces and washed sequentially with 50% acetonitrile (ACN) in 25 mM TEABC, 25 mM TEABC, and 100% ACN to dehydrate the gel. After vacuum drying, the gel was resuspended in 25 mM TEABC with trypsin (trypsin:protein = 1:10, weight ratio) for incubation at 37°C for 12 h. Peptides were extracted and resuspended in 100 mM TEABC to determine the peptide concentration by using the BCA assay.

**Tandem mass tag labeling**

Forty-one microliter of anhydrous ACN was added to each vial of 10-plexed tandem mass tag (TMT), which contained 0.8 mg in weight (Thermo Fisher Scientific). TMT_126_ was labeled with pooled reference from all patients and controls. TMT_127_ to TMT_131_ were labeled with serum peptides from patients with FM and healthy controls. For each sample, 4.1 µL of the TMT reagent was added to 5 µg of peptide and incubated for 1 h at room temperature. One microliter of 5% hydroxylamine was then added and incubated for 15 min to quench the reaction. In this study, five batches of 10-plex TMT experiments were used for 20 patients and 20 healthy controls. In each batch, 9 TMT-labeled samples were combined for high-pH reversed phase (Hp-RP) StageTip fractionation.

**High-pH RP StageTip fractionation**

To prepare Hp-RP StageTips, the C8 membrane was properly inserted into Gilson 200-µL pipet tips used as frits. Five milligram of C18-AQ beads (5 µm) was packed into the StageTips, washed, and conditioned adequately with centrifugation at 1,500 ×*g* for 2 min. Twenty microgram of TMT-labeled peptides was redissolved in 200 mM ammonium formate (pH 10) and transferred into the StageTips; this was followed by centrifugation at 1,500 ×*g* for 2 min to elute peptides in six reversed-phase fractions with increasing ACN concentrations. The eluted peptides were vacuum dried and stored at −80°C for further analysis.

**LC–MS/MS analysis**

All RP fractions were analyzed using LTQ Orbitrap Fusion mass spectrometers (Thermo Fisher Scientific, Bremen, Germany) equipped with the Dionex Ultimate 3000 nanoLC system (Thermo Scientific). Hp-RP fractions were resuspended in 0.1% formic acid (FA) and loaded onto a C18 column measuring 25 cm × 75 mm (Acclaim PepMap® RSLC, Thermo Scientific^TM^) equipped with a NanoSpray interface. Peptides were eluted at a flow rate of 0.3 µL/min by using a gradient from 5%–45% ACN in 0.1% FA with slight modifications for each RP fraction. The LTQ Orbitrap Fusion was operated in a data-dependent acquisition mode to sequentially isolate the 15 most intense ions (isolation window of 0.7 Da) for higher energy collision dissociation with collision energy of 40%. The previously selected ions were dynamically excluded for 15 s. The mass spectrometry survey scan (mass-to-charge ratio [*m–z*] 350–1600) was operated in orbitrap with a resolution of 120,000, max ion time of 50 ms, and automatic gain control of 4E5. The tandem mass spectrometry (MS/MS) scan (*m–z* 110–2000) was operated in orbitrap with resolution of 60,000, max ion time of 100 ms, and automatic gain control of 1E5.

**Proteome identification and quantitation**

The protein identification and quantification were performed using Proteome Discoverer (PD, version 2.1, Thermo Fisher Scientific). The MS/MS raw files were searched against the SwissProt human protein sequence database (released 2016_05) using Mascot engine in PD. The mass tolerances for precursor and fragment ions were 20 ppm and 0.1 Da, respectively. Tryptic peptides with a maximum of two missed cleavages were allowed. Methylthio (Cys) was set as a fixed modification; acetyl (protein N-term), oxidation (Met), deamidation (Asn and Gln), and TMT (N-term, Lys) were set as dynamic modifications. To ensure high confidence in identification, a 1% false discovery rate was applied in peptide spectra match (PSM), peptide and protein levels (P < 0.05) using percolator scoring. Protein and peptide quantitation were obtained based on the reported S–N ratio with a threshold of 10, and the co-isolation threshold was 50%. Only unique peptides were used to determine the protein abundances. For proteome quantitation, the protein abundances in each sample and control reference were normalized with respect to the total protein abundance of each sample and control reference. The protein abundance ratio was defined as scaled protein abundance in the sample divided by one in the corresponding control reference.


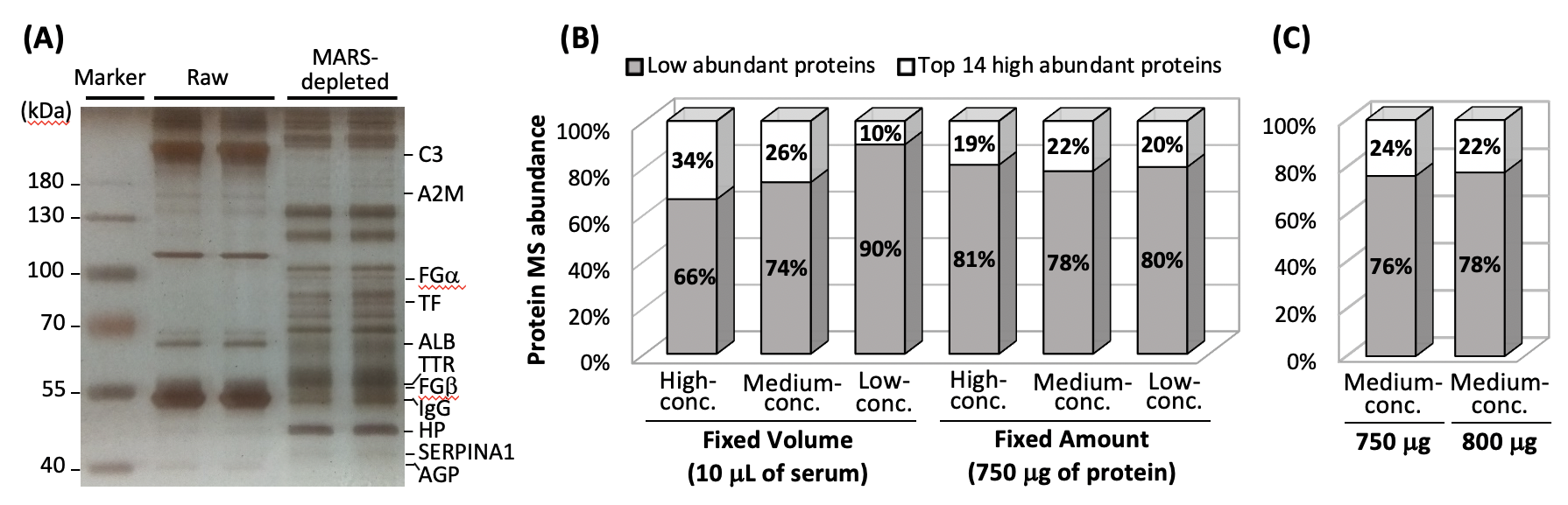


**Supplementary Figure S1.** Optimization of depletion of high-abundant proteins by MARS Hu-14. (A) SDS-PAGE analysis of raw and MARS-depleted serum. (B) LC–MS/MS quantitation analysis of abundance for the top 14 high-abundance proteins and other low-abundance proteins after MARS depletion with equal volumes of serum or equal amounts of serum proteins. (C) Loading amounts of 750 mg and 800 mg serum proteins were evaluated for MARS depletion.

**Supplementary Table S1. Pathway analysis of significant candidate proteins.**

| **IPA Canonical Pathways** | **−log(P value)** | **Ratio** | **z-score** | **Molecules** |
| --- | --- | --- | --- | --- |
| Acute Phase Response Signaling | 8.35 | 0.0335 | - | C4A/C4B, APCS, FGA, SAA4, IL1RAP, F2 |
| LXR–RXR Activation | 5.61 | 0.0331 | −1 | C4A/C4B, FGA, SAA4, IL1RAP |
| Extrinsic Prothrombin Activation Pathway | 4.13 | 0.125 | - | FGA, F2 |
| FXR–RXR Activation | 3.86 | 0.0238 | - | C4A/C4B, SAA4, FGA |
| Coagulation System | 3.44 | 0.0571 | - | FGA, F2 |
| Intrinsic Prothrombin Activation Pathway | 3.28 | 0.0476 | - | FGA, F2 |
| Role of Tissue Factor in Cancer | 2.27 | 0.0148 | - | FGA, F2 |
| Clathrin-mediated Endocytosis Signaling | 1.91 | 0.00962 | - | SAA4, F2 |
| Actin Cytoskeleton Signaling | 1.82 | 0.00855 | - | PFN1, F2 |
| Inhibition of Angiogenesis by TSP1 | 1.56 | 0.0294 | - | THBS1 |
| Role of Macrophages, Fibroblasts and Endothelial Cells in Rheumatoid Arthritis | 1.54 | 0.00612 | - | IL1RAP, FCGR3A/FCGR3B |
| Synaptogenesis Signaling Pathway | 1.54 | 0.00612 | - | THBS1, THBS2 |
| Complement System | 1.53 | 0.027 | - | C4A/C4B |
| Inhibition of Matrix Metalloproteases | 1.5 | 0.0256 | - | THBS2 |
| PCP Pathway | 1.31 | 0.0164 | - | PFN1 |

**Supplementary Table S2. Upstream regulator analysis of significant candidate proteins.**

| **Upstream Regulator** | **Molecule Type** | **Activation z-score** | **P value of overlap** | **Target molecules in dataset** |
| --- | --- | --- | --- | --- |
| IL6 | cytokine | −1.282 | 1.19E-05 | APCS, FGA, GP1BA, GP5, SAA4, THBS1 |
| lipopolysaccharide | chemical drug | −0.374 | 0.00832 | APCS, F2, IL1RAP, THBS1, THBS2 |
| MYC | transcription regulator | −0.152 | 0.00104 | F2, GP1BA, IL1RAP, THBS1, THBS2 |
| TNF | cytokine | 0.152 | 0.0392 | APCS, GP1BA, THBS1, THBS2 |
| TGFB1 | growth factor | 0.328 | 0.00787 | F2, FCGR3A/FCGR3B, FGA, THBS1, THBS2 |

**CONSORT of the study**

**Supplementary Figure S2. The CONSRT flow diagram of present study. The reasons to exclude patients were concurrent malignancy (n=1) and concurrent major rheumatic disease (n=4).**
